# Cardiac Lymphoma: A Rare Cause of Acute Heart Failure with Restrictive Physiology

**DOI:** 10.5935/abc.20180015

**Published:** 2018-02

**Authors:** Fernando Garagoli, Ezequiel Guzzetti, Ezequiel Lillo, Luciano Lucas, César Belziti

**Affiliations:** Hospital Italiano de Buenos Aires, Buenos Aires - Argentina

**Keywords:** Cardiovascular Diseases, Lymphoma, Heart Failure / physiopathology, Magnetic Resonance Imaging

**Figure 1:**
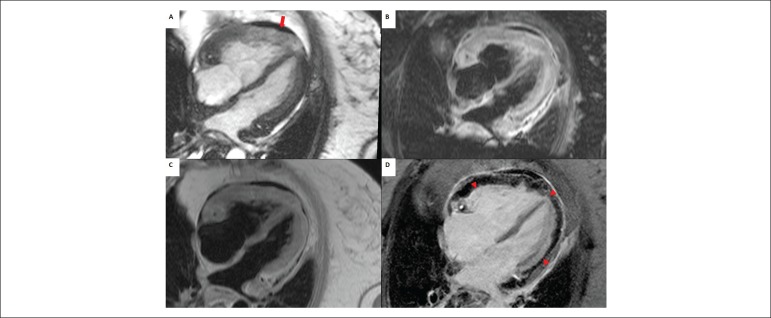
a) Steady -state free precession (SSFP)-cine imaging showing an ill-defined, heterogeneous myocardial mass involving all cardiac chambers, particularly the right ventricle wall and right atrioventricular groove, as well as moderate pericardial effusion (solid arrow). b) T2-weighted magnetic resonance imaging showing hyperenhancement of the mass, compatible with edema. c) T1-weighted sequence showing isointensity of the heterogeneous mass. d) T1-weighted inversion recovery showing late gadolinium enhancement of the myocardium (compatible with myocardial fibrosis) and hypoenhancement of the mass, marking the limit between myocardium and tumor (arrowheads).

A 74-year-old woman with a history of membranous glomerulonephritis and a recent diagnosis of mediastinal adenopathy was admitted to the emergency department with acute heart failure. She complained of progressive dyspnea and weakness in the last week. Physical examination revealed hypotension, tachypnea, jugular vein distention, and desaturation. The most relevant laboratory findings were: anemia, lymphocytopenia, lactic acidosis, and increased lactate dehydrogenase. An electrocardiogram showed rapid atrial fibrillation and low-voltage QRS complexes. An echocardiogram revealed severe pericardial effusion and diffuse heterogeneous thickening of the ventricular and atrial walls. The patient required mechanical ventilation and inotropic support. Therapeutic pericardiocentesis was performed without clinical improvement. Cardiovascular magnetic resonance imaging (CMR) showed septal bounce (compatible with restrictive physiology) and a heterogeneous isointense mass surrounding the ventricular and atrial walls with late gadolinium enhancement of the myocardium and hypoenhancement of the tumor (Figure 1), compatible with primary cardiac lymphoma. A diagnosis of large B-cell lymphoma was confirmed by flow cytometry of the pericardial fluid. The patient died before starting chemotherapic treatment.

Secondary involvement of the myocardium in patients with systemic lymphoma is relatively frequent (around 30% in disseminated non-Hodgkin lymphoma) whereas primary cardiac lymphoma is rare (1-2%). We present a case of acute heart failure with restrictive physiology secondary to cardiac lymphoma. In our experience, CMR was key to the final diagnosis.

